# An Inter- and Intra-Subject Transfer Calibration Scheme for Improving Feedback Performance of Sensorimotor Rhythm-Based BCI Rehabilitation

**DOI:** 10.3389/fnins.2020.629572

**Published:** 2021-01-28

**Authors:** Lei Cao, Shugeng Chen, Jie Jia, Chunjiang Fan, Haoran Wang, Zhixiong Xu

**Affiliations:** ^1^Department of Artificial Intelligence, Shanghai Maritime University, Shanghai, China; ^2^Department of Rehabilitation Medicine, Huashan Hospital, Fudan University, Shanghai, China; ^3^Wuxi Rehabilitation Hospital, Wuxi, China; ^4^Department of Computer Science and Technology, Tongji University, Shanghai, China

**Keywords:** BCI, transfer learning, rehabilitation, stroke, classifier calibration

## Abstract

The Brain Computer Interface (BCI) system is a typical neurophysiological application which helps paralyzed patients with human-machine communication. Stroke patients with motor disabilities are able to perform BCI tasks for clinical rehabilitation. This paper proposes an effective scheme of transfer calibration for BCI rehabilitation. The inter- and intra-subject transfer learning approaches can improve the low-precision classification performance for experimental feedback. The results imply that the systematical scheme is positive in increasing the confidence of voluntary training for stroke patients. In addition, it also reduces the time consumption of classifier calibration.

## 1. Introduction

Brain-computer interface (BCI) is developed as an extrinsic pathway for human-machine interaction in a reliable way (Birbaumer, 2006). It is effective for the disabled to control external devices by neural activities (Buch et al., 2008). Stroke patients with motor disabilities in particular, are able to perform BCI tasks for clinical rehabilitation (Meng et al., 2016). In this treatment, sensorimotor rhythm changes are used as neurological modulation for active intervention (Mane et al., 2019).

During rehabilitation, the patients are requested to attempt or to imagine performing a movement. Then, motor attempt (MA) or motor imagery (MI)-BCI systems will output a synchronized sensory biofeedback (e.g., robotic arm recovery) by a trained classifier based on a prior dataset (Pillette et al., 2020). In the intervention, the functional motor is significantly enabled by neurophysiological activity (Xu et al., 2014). This is an ongoing process of brain plasticity and functional recovery (Remsik et al., 2019). Recent studies have reported the improvement of limb movement for stroke patients using long-term sensorimotor rhythm (SMR)-BCI interventions (Ramos-Murguialday et al., 2013; Pichiorri et al., 2015; Bundy et al., 2017).

Nevertheless, BCI rehabilitation is limited by poor-efficiency recognition algorithms and model-personalized variability (Grosse-Wentrup et al., 2011). Relevant work has proved that BCI decoding accuracy was insufficient for rehabilitation outcomes (Mane et al., 2020). Moreover, the failures of BCI feedback also reduce the confidence of trainees subtly (Foong et al., 2019). Hence, various improvements of pattern recognition and model calibration should be made to enhance SMR-BCI performance in the clinical application.

Conventionally, SMR features are effectively extracted by a time-frequency analysis for the healthy (Pfurtscheller and Da Silva, 1999). For instance, the event-related desynchronization (ERD) amplitudes oscillated in the μ rhythm was detected during the motor imagery task for pattern recognition (Huang et al., 2012; Saha et al., 2017). Furthermore, the common spatial pattern (CSP) algorithm was proposed for feature extracting in the spatial domain (Wang et al., 2006; Arvaneh et al., 2013). It was efficient for mining the significant difference of two-type motor tasks. However, the degeneration of neural activation due to post stroke, has a negative impact on BCI performance (Shu et al., 2018). Thus, the signal characteristics are much lower than those of healthy individuals during motor tasks (De Vries et al., 2013; Caria et al., 2020). Therefore, increasing the precision of SMR-BCI using a mathematical methods is meaningful for BCI intervention.

To solve this problem, transfer learning (TL), which applies the dataset in source domains for compensating insufficient labeled data in a target domain, has been proposed for MI-BCIs (Samek et al., 2013b; Azab et al., 2018). This technology is developed in several ways, such as instance selection (Wu, 2016; Hossain et al., 2018), feature calibration (Samek et al., 2013a; Zhao et al., 2019) and classification domains (Vidaurre et al., 2010; He and Wu, 2019). For instance, for selection, active learning is typically presented for selecting training data from intra- or inter-subject labeled trials (Hossain et al., 2018). The target of this approach is to increase the informative trials of the new subject by adding sufficient existing labeled trials that were close to prior dataset. In the feature domain, transfer calibration approaches mainly concentrate on regulating the covariance matrix estimation and optimization function for improving the performance of CSP models. For example, researchers regularized the CSP filter by the average of the common feature space from other subjects (Kang et al., 2009). Moreover, the efficiency of domain adaptation has been verified for MI-BCI in the classification domain (Vidaurre et al., 2010). Kobler et al. constructed a Restricted Boltzmann Machine (RBM) based on public baseline data and applied it for the MI-BCI task (Kobler and Scherer, 2016). Recently, multi-task learning has been presented in the relevant experiment (Jayaram et al., 2016; Gao et al., 2019), where the weight parameters of inter-subjective classifiers were learned jointly for minimizing the dissimilarities between these existing classification models and the target model. However, BCIs controlled by these approaches have been proven to be only valid for healthy individuals. None of them are experimentally evaluated for BCI rehabilitation.

This paper first proposes a transfer calibration scheme to improve the rehabilitation outcomes of SMR-BCI. First, we utilize a transfer learning algorithm, whose effects have been verified in the task of MI-BCI (Azab et al., 2019), to validate the reliability for stroke patients. Then, we discuss the respective applicability between intra- and inter- subjective conditions. The results show that our proposed approaches improved the low-precision classification performance. Accordingly, we generalize the scheme of transfer calibration for SMR-BCI intervention. This methodology applies for other transfer learning algorithms to increase the precision of BCI feedback.

## 2. Materials and Methods

### 2.1. Experimental Paradigm and Subjects

Seven stroke patients aged between 30 and 65, recruited from the Department of Rehabilitation Medicine of Huashan Hospital participated in our experiments (Table 1). All of them were naive to BCI and provided consent to be involved in the study. The inclusion criteria for this study were as follows: (1) unilateral motor dysfunction diagnosed by computer tomography or magnetic resonance imaging (MRI); (2) first onset stroke patient; (3) the time since stroke onset was more than 4 weeks and less than 6 months; (4) the assessment of cognitive functions: Mini-Mental State Examination score >25. The exclusion criteria were listed below: (1) unstable medical conditions; (2) severe vision problems; (3) the intervention treatment by other brain stimulations during the study period.

**Table 1:** The baseline clinical characteristics of participants.

**Patients**	**Gender**	**Affected hand**	**Type of injury**	**Time since injury (m)**	**Site of injury**
S1	Male	Right	Ischemia	5	Left, basal ganglia
S2	Male	Left	Hemorrhage	4	Right, basal ganglia
S3	Male	Right	Hemorrhage	1	Left, basal ganglia
S4	Male	Right	Ischemia	1	Left, paracele
S5	Male	Right	Ischemia	3	Left, basal ganglia
S6	Male	Right	Ischemia	5	Left, paracele, basal ganglia
S7	Male	Left	Ischemia	3	Right, basal ganglia

**Figure 1:**
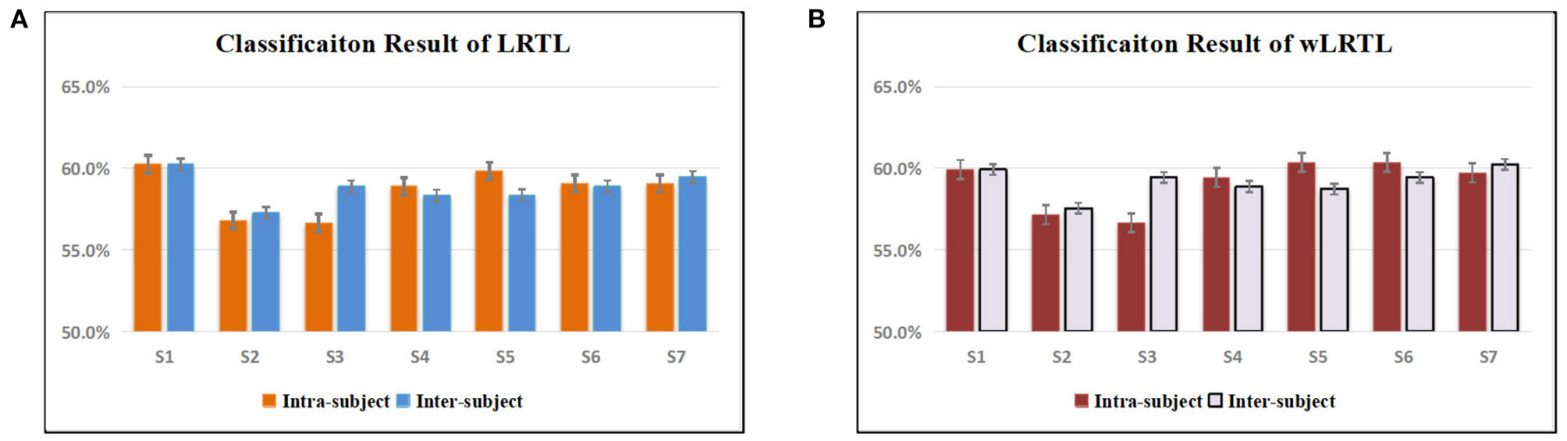
**(A)** Experimental procedures of two different mental tasks. In the task of MA, patients were required to attempt motion of wrist extension with affected hands continually, but not to have compensatory movements. In the other task of IS, they need not do anything for a rest. **(B)** Overview of BCI control paradigm.

In the experiment, BCI intervention was performed in three sessions a week for each patient. And it lasted 1 month, with a total of 12 sessions. One session contained three runs, each run had 30 trials for each mental task (motor attempt or idle state) performed in a random order. Subjects underwent two experimental tasks. In the task of motor attempt (MA), patients were required to attempt motion of wrist extension with affected hands continually, but not to have compensatory movements. In the other task of idle state (IS), they needed to do nothing but rest (Figure 1A).

The experimental paradigm is shown in Figure 1B. In one trial, the duration time was about 11 s. The patient was asked to sit in front of a computer screen, with arms resting on a desk. A white arrow presented on the center of the screen from 0 to 3 s. The patient was instructed to keep still and rest. Then, an alternative indicator (a red square or a red rectangle) was displayed in the center of the screen, representing a task (MA or IS). After the command disappeared, the subject was required to perform the corresponding task for 5 s until the white cross disappeared. Finally, the rest interval was adopted randomly to relax.

### 2.2. EEG Recording and Signal Preprocessing

EEG were collected using 32 channels consisting of Ag/AgCl electrode of EEG cap (actiCAP; Brain Products, Germany) according to the configuration of 10-C20 International System. A bio-signal amplifier (Brain Products) was used for acquiring the signals. The unilateral reference electrode was located in the right mastoid process, and the ground electrode was located in the forehead. The other 31 channels (FP1, FZ, F3, F7, FT9, FC5, FC1, C3, T7, TP9, CP5, CP1, PZ, P3, P7, O1, O2, P4, P8, TP10, CP6, CP2, CZ, C4, T8, FT10, FC6, FC2, F4, F8, FP2) were used for calculation. Electrode impedances were kept below 5 kΩ. The signals were amplified, digitalized with a sample rate of 200 Hz, and bandpass-filtered between 1 and 35 Hz.

### 2.3. Evaluation of BCI Performance

BCI performance was evaluated by classification accuracy for two mental tasks. As shown in the figure of experimental paradigm, the subject conducted the mental task from 4.5 s to 9.5 s. Hence, EEG signals were extracted from [4.5 9.5] s of each single trial. And the features were filtered with the common spatial pattern (CSP) method, whose log-variance of the first and last three components were selected as output vectors. Then, a transfer learning scheme was used to improve the classification efficiency. The Logistic Regression (LR) -based classifier was used as the baseline approach. Its classification parameters *w*_*t*_ were calibrated using the following function,

(1) $$\begin{array}{l} L\left(w_{t}\right)=\underset{w_{t}}{\min}\left(\overset{i=1}{\underset{n_{t}}{\sum }}H\left(w_{t};l_{t}^{i},f_{t}^{i}\right)+\lambda_{t}\|w_{t}{\|}_{2}^{2}\right), \end{array}$$

where *H*, *f*_*t*_, *l*_*t*_ and ||.||_2_ denote the cross-entropy, feature vectors extracted from the EEG dataset, label vector, and 2-norm functions, respectively. Conventionally, the parameters were supposed be trained by large prior data. Transfer learning algorithms had been proposed for improving the efficiency of parameter calibration of new subjects without subject-specific data (Hossain et al., 2018). In the framework of logistic regression -based transfer learning (LRTL), a regularization term penalizing dissimilarities *R*_*t*_ was used for transferring the prior distribution of the existing classification parameters into the calibration of the present training of new target subjects or sessions. In this opinion, the classification parameters were calculated as follows,

(2) $$\begin{array}{l} L^{\prime }\left(w_{t}\right)=\underset{w_{t}}{\min}\left(\overset{i=1}{\underset{n_{t}}{\sum }}H\left(w_{t};l_{i}^{t},l_{i}^{f}\right)+\lambda_{t}R_{t}\left(w_{t}\right)\right), \end{array}$$

and the *R*_*t*_ was decided by estimating the similarity between feature distribution of existing models and that of current few training data,

(3) $$\begin{array}{l} R_{t}\left(w_{t}\right)=0.5\left[{\left(w_{t}-\mu \right)}^{T}\Sigma_{t}^{-1}\left(w_{t}-\mu \right)+\log\left(\left|\Sigma_{t}\right|\right)\right], \end{array}$$

where μ and Σ_*t*_ were, respectively, obtained as

(4) $$\begin{array}{l} \mu =\frac{1}{n}\overset{n}{\underset{t^{\prime }=1}{\sum }}w_{t^{\prime }}, \end{array}$$

(5) $$\begin{array}{l} \Sigma_{t}=\frac{diag\left(\Sigma_{t^{\prime }=1}^{n}\left(w_{t^{\prime }}-\mu \right){\left(w_{t^{\prime }}-\mu \right)}^{T}\right)}{trace\left(\Sigma_{t^{\prime }=1}^{n}\left(w_{t^{\prime }}-\mu \right){\left(w_{t^{\prime }}-\mu \right)}^{T}\right)}. \end{array}$$

Here, T denoted transpose of the matrix, the functions of *diag* and *trace* were defined as the diagonal elements and the sum of the diagonal elements of a matrix. Furthermore, Kullback-Leibler (KL) divergence was added to solve the problem of weight distribution between existing models and the target model. It was supposed to give larger weights to more similar distributions and smaller weights to less similar distributions. KL divergence of two EEG sets (*E*_0_, *E*_1_) were represented as the following form,

(6) $$\begin{array}{l} KL\left[E_{0}\|E_{1}\right]=0.5[{\left(\mu_{1}-\mu_{0}\right)}^{T}\Sigma_{1}^{-1}\left(\mu_{1}-\mu_{0}\right)+trace\left(\Sigma_{1}^{-1}\Sigma_{0}\right)\\ \;-ln\left(\frac{det\left(\Sigma_{0}\right)}{det\left(\Sigma_{1}\right)}\right)-K], \end{array}$$

(7) $$\begin{array}{l} \Sigma_{t_{w}}=\frac{diag\left(\Sigma_{t^{\prime }=1}^{n}\left(\alpha_{t^{\prime }}w_{t^{\prime }}-\mu_{t}\right){\left(\alpha_{t^{\prime }}w_{t^{\prime }}-\mu_{t}\right)}^{T}\right)}{trace\left(\Sigma_{t^{\prime }=1}^{n}\left(\alpha_{t^{\prime }}w_{t^{\prime }}-\mu_{t}\right){\left(\alpha_{t^{\prime }}w_{t^{\prime }}-\mu_{t}\right)}^{T}\right)}, \end{array}$$

where *det* and K denoted the determinant function and the dimension of the data, respectively. Thus, the Σ_*t*_ was updated as below,

(8) $$\begin{array}{l} \alpha_{t^{\prime }}=\frac{\left(1/{\left(KL\left[E_{s},E_{t^{\prime }}\right]+\varepsilon \right)}^{4}\right)}{\left(1/{\left(KL\left[E_{s},E_{i}\right]+\varepsilon \right)}^{4}\right)}, \end{array}$$

where α and μ was computed as

(9) $$\begin{array}{l} \mu_{t}=\frac{1}{n}\overset{n}{\underset{t=1}{\sum }}\alpha_{t^{\prime }}w_{t}. \end{array}$$

Here, the divergence was calculated by averaging the KL divergences calculated for each class separately. The details of weighted LRTL (wLRTL) approach and the above other algorithms can be reviewed in Azab et al. (2019).

In this study, we discussed the transfer scheme for several subjective conditions of prior knowledge. Inter- and Intra-subject transfer learning approaches were both used to evaluate the methodological effectiveness. Inter-subject transfer calibration trained this classifier with prior experimental trials from other subjects while intra-subject transfer calibration performed this work using its own existing dataset. The target of our research was to find which kind of transfer strategies could be made to improve the online single-trial accuracy of BCI rehabilitation. The right bio-feedback (e.g., robotic arm) was able to raise the subjective confidence and patience to improve the therapeutic effect.

In this experiment, the performances of first sessions were unavailable for intra-subject transfer learning algorithms on account of no prior dataset. We used the sequential collecting of source data for transfer calibration. That meant that the target session (e.g., Session 5) would be trained by all prior collections (e.g., Session 1, Session 2, Session 3, Session 4). It was consistent with the real condition of model training. Meanwhile, the collection of source data was picked from all other subjects under the inter-subject condition. Specifically, the dataset of each subject which obtained the best performance in all sessions was used for transfer learning. Moreover, 5-fold cross validation was conducted for each approach. All 31 channels of the EEG data were selected for pattern classification. The 45 trials of MA and IS tasks (45 trials per class) were randomly divided into five sets. Four sets were used to train the classifier and the other set was tested to evaluate the performance.

## 3. Results

### 3.1. The Experimental Performance of Intra-Subject Based Transfer Learning Approach

**Figure 2:**
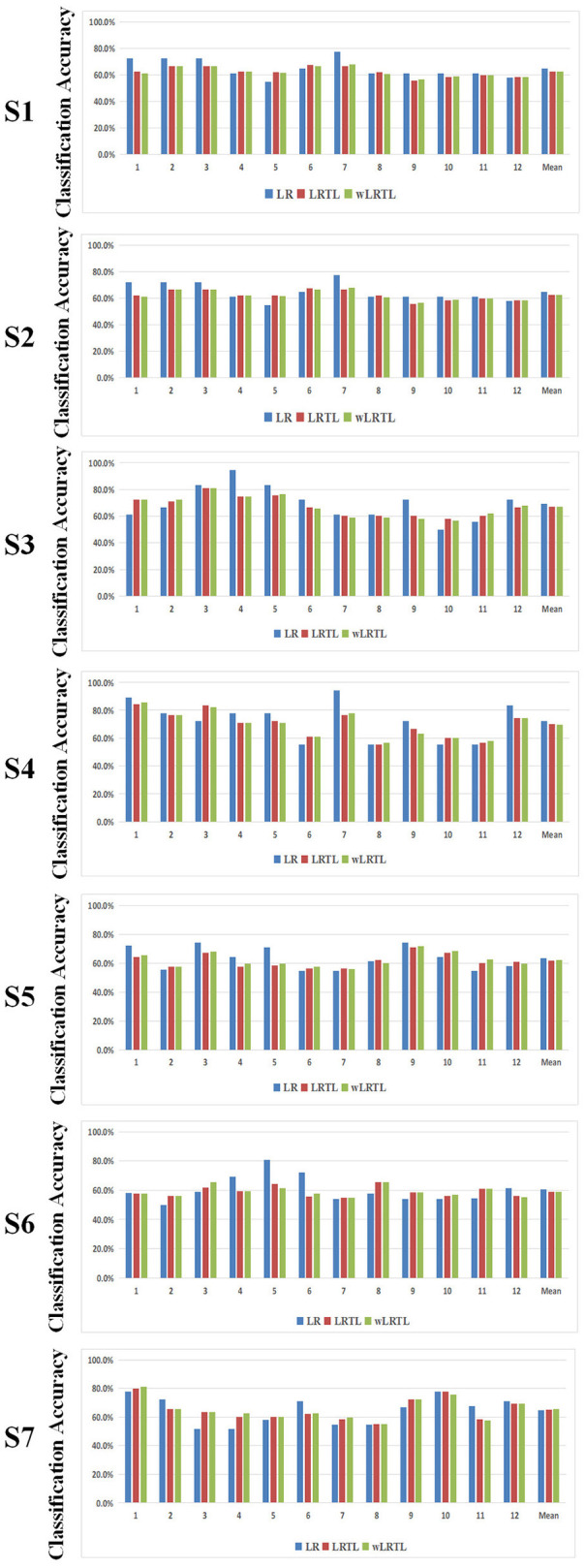
The classification results of transfer learning algorithms and baseline algorithm under the intra-subject condition. Specially, the accuracies were missing for LRTL and wLRTL. Because the new subject had no experience of relevant tasks. Hence, the transfer calibration was not performed without prior dataset under this condition.

In our study, the precision of pattern recognition was considered as the most important index for BCI rehabilitation. Figure 2 lists the classification results of the above intra-subject transfer learning approaches (LRTL, wLRTL), as well as the baseline approach (LR). The average classification accuracies of all patients were higher than 60% for three different algorithms, except for P6. And it was indicated that the pattern of motor attempt could be distinctive from that of an idle state without motor attempt. However, a paired *t*-test with Bonferroni correction showed that no discriminatory differences were presented between transfer learning approaches and the baseline approach (LR vs. LRTL: *p* = 0.0277; LR vs. wLRTL: *p* = 0.0613; LRTL vs. wLRTL: *p* = 0.6085). This result suggests that transfer calibration did not significantly improve the BCI performance.

Furthermore, we analyzed the performance of low-precision (≤ 60%) sessions for all patients (Table 2). Paired *t*-test with Bonferroni correction showed that the classification results of transfer learning approaches were significantly greater than that of the baseline approach (LR vs. LRTL: *p* = 0.0001; LR vs. wLRTL: *p* = 0.0001; LRTL vs. wLRTL: *p* = 0.0563). It was meaningfully revealed that transfer calibration could improve the BCI performance induced by poor model training.

**Table 2:** The average accuracy of low-precision sessions (≤ 60%) for transfer learning algorithms (i.e., LRTL, wLRTL) and baseline algorithm (i.e., LR) under the intra-subject condition.

**Methods**	**S1**	**S2**	**S3**	**S4**	**S5**	**S6**	**S7**
LR (%)	56.5	53.6	52.8	55.6	55.6	54.7	54.2
LRTL (%)	60.2	56.8	56.7	58.9	58.2	59.8	59.1
wLRTL (%)	59.9	57.2	56.7	59.4	58.6	60.4	59.7

*The accuracies of transfer learning approaches were higher than that of baseline approach*.

### 3.2. Inter-Subject Based Transfer Learning Approach for MI-Based BCI Rehabilitation

Similarly, Table 3 lists the classification results of all three algorithms under the inter-subject condition. Paired *t*-test with Bonferroni correction showed that no discriminative differences were presented between transfer learning approaches and the baseline approach (LR vs. LRTL: *p* = 0.0488; LR vs. wLRTL: *p* = 0.1207; LRTL vs. wLRTL: *p* = 0.1744). This result suggested that the non-significance was consistent with those under the intra-subject condition.

**Table 3:** The average accuracy of low-precision sessions (≤ 60%) for transfer learning algorithms (i.e., LRTL, wLRTL) and baseline algorithm (i.e., LR) under the inter-subject condition.

**Methods**	**S1**	**S2**	**S3**	**S4**	**S5**	**S6**	**S7**
LR (%)	56.5	52.9	52.8	55.6	55.6	55.1	54.2
LRTL (%)	60.2	57.3	58.9	58.3	58.3	58.9	59.5
wLRTL (%)	59.9	57.6	59.4	58.9	58.7	59.4	60.2

*The accuracies of transfer learning approaches were higher than that of baseline approach*.

**Figure 3:**
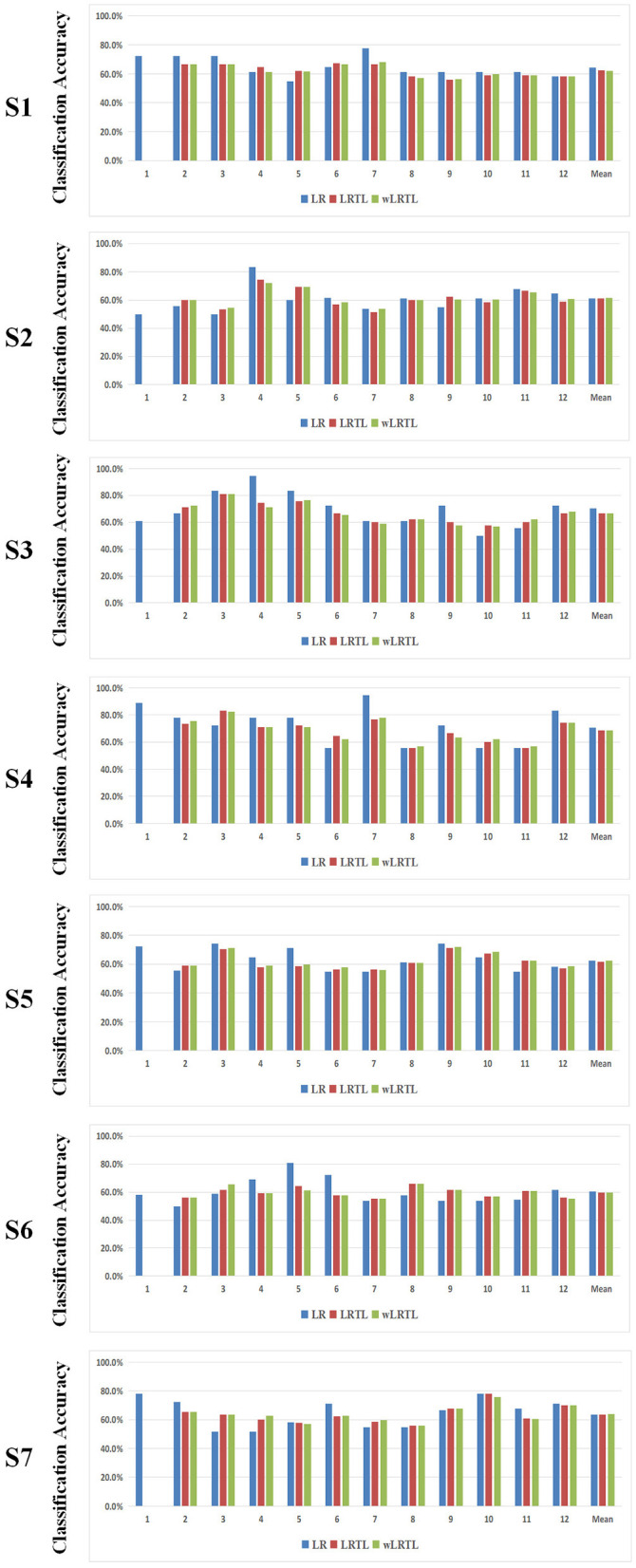
The classification results of transfer learning algorithms and baseline algorithm under the inter-subject condition.

Additionally, low-precision sessions were extracted for further analysis (Figure 3). In this case, the statistical analysis indicated that the accuracies of transfer learning approaches were significantly higher than that of the baseline approach (LR vs. LRTL: *p* = 0.0001; LR vs. wLRTL: *p* = 0.0001; LRTL vs. wLRTL: *p* = 0.0210). It was revealed that transfer calibration improved the BCI performance under the inter-subject condition.

## 4. Discussion

This study proposed a novel transfer calibration scheme to improve low-precision performance for BCI rehabilitation. This scheme of transfer learning could be used for new subjects without the training dataset, as well as replacing the poor training model–whose accuracy was close to the chance level. Cerebral activities were also observed to clarify the benefit of transfer calibration for feature selection.

### 4.1. Improvement of Low-Precision Performance for BCI Rehabilitation

As we know, the critical issue of BCI rehabilitation revolves around how to promote the biofeedback effect for active intervention (Ko et al., 2019). It was positive for rehabilitation outcomes, enhancing cortical activity for neural recovery, and increasing confidences of voluntary training (Zhang et al., 2020). Hence, the biofeedback of low-precision trials was negative for patients. Furthermore, most of the sessions used by the baseline classifier achieved the effective performance (>60%) for each patient, except for S6. It was indicated that very few non-effective results of experimental sessions were unreliable for evaluating this neural treatment. Improved performance of transfer calibration could eliminate the confusion caused by the precision fluctuation.

**Figure 4:**
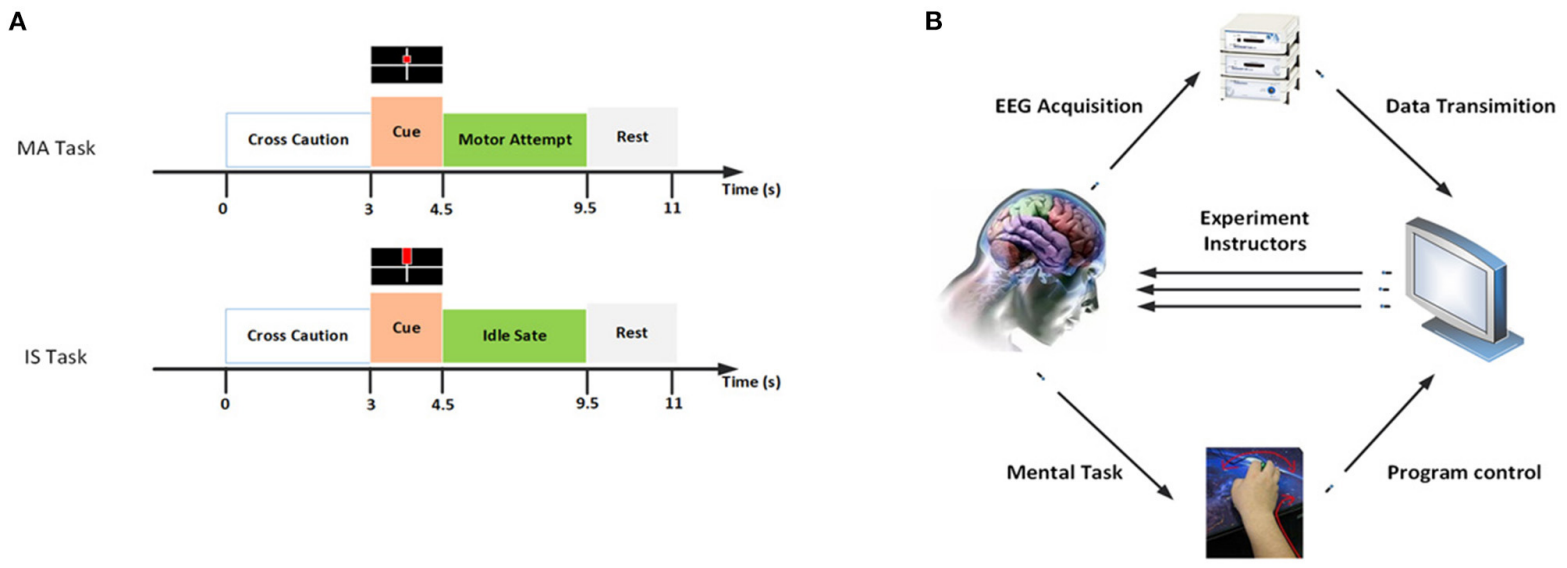
**(A)** The comparison of low-precision classification results for LRTL algorithm between intra-subject and inter-subject conditions. **(B)** The comparison of low-precision classification results for wLRTL algorithm between intra-subject and inter-subject conditions.

Moreover, we presented the comparison of transfer learning algorithms between inter-subject (IERS) and intra-subject (IRAS) conditions (Figure 4). Statistical analysis was used to compare the classification accuracies among transfer conditions (LRTL: IERS vs. IRAS: *p* = 0.3138; wLRTL: IERS vs. IRAS: *p* = 0.3501;). This result suggested that both of them were reliable for improving low-precision performance of SMR-BCI tasks. However, the advantage of inter-subject transfer calibration was employed for new subjects without training.

The fluctuation of classification for LR resulted from the differences of brain activity between consecutive sessions. It was deduced that changes of cerebral activities were caused by neural self-rehabilitation for patients. As a result, BCI-based intervention was effective for stroke rehabilitation to some extent. Nevertheless, the methodology needs to be further clarified for high-efficiency treatment.

The efficiency of transfer learning has been verified by motor imagery -based BCI tasks for healthy people. However, cerebral impairment of stroke patients would influence the training effect due to the weak neural activities. Therefore, our classification result was lower than those of the above state-of-art BCI systems (Azab et al., 2019, 2020; He and Wu, 2019). Nevertheless, the improvement of low-precision performance was conductive to treatment for the impatient. Compared to the feedback of the random level, the subject was subjectively motivated by positive feedback of right detection. It is meaningful for long-term continuous rehabilitation.

### 4.2. Transfer Calibration Scheme for SMR-Based BCI Intervention

**Figure 5:**
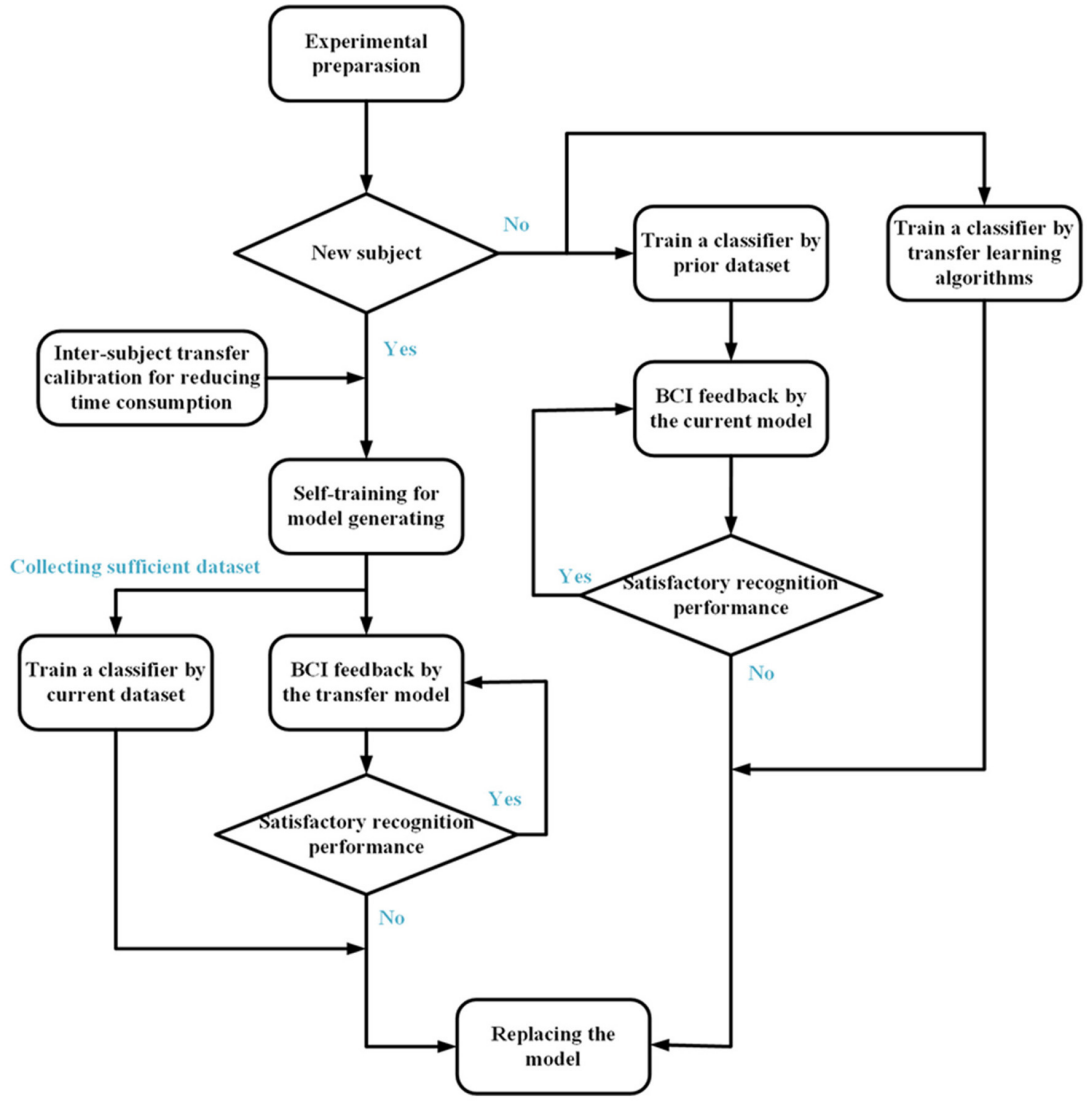
The transfer calibration scheme of classifier model selection for SMR-BCI rehabilitation. For a new subject, intra- or inter-subject transfer calibration could be used for reducing the calibration time. Meanwhile, we could train another model when sufficient trials were finished in the task. If the precision of current using model was superior to that of the spare model, the alternative could be automatically performed by control system.

In our study, an available scheme of transfer calibration was proposed for model selection in the online task of SMR-BCI rehabilitation (Figure 5). We summarized several rules as stated below: (1) Instant self-training was necessary for new subjects. The classification model based on current dataset was reliable for BCI tasks. (2) If the patient was frustrated by tedious training, intra- or inter-subject transfer calibration could be used to reduce the calibration time. (3) Furthermore, we could train another model when sufficient trials were finished in the task. If the precision of current model was superior to prior transfer model, the alternative could be automatically performed by our control system. (4) If the model based on the current training dataset performed poorly on the experiment, intra- or inter-subject transfer calibration was worth trying in order to replace the under-performing model. (5) For transfer calibration, the volume of EEG data was a crucial factor for model selection between intra-subject and inter-subject conditions. Sufficient training data was an essential precondition for transfer calibration. Specifically, the only option for new subjects without prior experience was inter-subject transfer calibration.

This scheme of transfer calibration was feasible for improving the poor performance of SMR-BCI recognition. And it also reduced the time consumption of model calibration. It was inferred that this scheme was suitable for other transfer learning algorithms.

### 4.3. Limitation of Current Work

In this study, some issues should be noted and considered in our future work. First, the scheme of transfer calibration needed to be verified for a large amount of stroke patients. It will be addressed in future studies. Second, these patients performed these experiments for 3 months. BCI training over longer time periods should be observed to evaluate performances of the patients in different stages of post-stroke time. Moreover, the number of electrodes was supposed to be reduced by data analysis. It could reduce the time consumption of BCI rehabilitation. Thus, future studies should be conducted to solve these problems to improve the performance of online SMR-BCI rehabilitation.

## 5. Conclusions

This paper proposed an effective scheme of transfer calibration for SMR-BCI rehabilitation. The inter- and intra-subject transfer learning approaches could improve the low-precision classification model for BCI feedback. The results imply that this systematical scheme is positive in increasing confidence of voluntary training for stroke patients. It also reduced the time consumption of model calibration.

## Data Availability Statement

The raw data supporting the conclusions of this article will be made available by the authors, without undue reservation.

## Ethics Statement

The studies involving human participants were reviewed and approved by Ethics Committee of Huashan Hospital. The patients/participants provided their written informed consent to participate in this study.

## Author Contributions

LC and HW designed the methodology of data process and performed the data analysis. LC and SC organized the data and wrote the manuscript. LC, ZX, CF, and JJ reviewed and edited the manuscript. All authors read and approved the submitted manuscript.

## Conflict of Interest

The authors declare that the research was conducted in the absence of any commercial or financial relationships that could be construed as a potential conflict of interest.
